# Barriers to Healthy Eating Among High School Youth in Rural Southern Appalachia

**DOI:** 10.13023/jah.0102.04

**Published:** 2019-07-06

**Authors:** Jodi L. Southerland, Taylor M. Dula, Deborah L. Slawson

**Affiliations:** East Tennessee State University; East Tennessee State University; East Tennessee State University

**Keywords:** Appalachia, diet, nutrition, child health, obesity, healthy food, youth

## Abstract

**Introduction:**

Diet and nutrition play an important role in a child’s health and reduce the risk of numerous health problems including obesity. Dietary habits can be difficult to modify in children, particularly in Appalachia, where access to affordable, healthy foods is limited.

**Purpose:**

To examine barriers to healthy eating among Appalachian youth.

**Methods:**

In 2013–2014, data were gathered via focus groups and interviews from parents, school personnel, and adolescents (N=99) in six counties across southern Appalachia. Data were analyzed using thematic network analysis. Analysis was completed in 2015.

**Results:**

Participants identified multiple barriers to healthy eating among adolescents. Barriers comprised three global themes: cultural norms, school-based nutrition policy and programming, and rurality. Within the individual and home environment, beliefs and practices, time management, and preference for unhealthy foods affected adolescents’ dietary behaviors. Schools faced challenges in terms of translating nutrition policy into practice, providing nutrition education, and engaging stakeholders. Limited socioeconomics and food deserts/swamps were community-level impacts.

**Implications:**

Participants discussed how adolescents’ eating behaviors were shaped by social, physical, and environmental factors (e.g., cultural norms, school-based nutrition policy/programming, and rurality). These findings provide important insights into barriers to healthy eating in this population and point to future directions for research and practice. For example, the findings can be used by school personnel to develop ecologic approaches to school-based nutrition programming in Appalachia. Student associations can also use these findings to actively participate in efforts to improve school food offerings.

## INTRODUCTION

Obesity has grown to epidemic proportions. Nearly one in three U.S. adolescents (30.4%) are overweight/obese, with rates higher in Appalachia (46.4%).^1^,^2^ Proper diet and nutrition play an important role in an adolescent’s health and reduce the risk of numerous health problems; whereas a poor diet can increase risk of health problems like obesity.^3^ Among adolescents, diets are largely composed of empty-calorie foods and few fruits and vegetables. Dietary habits can be difficult to modify in adolescents, particularly in Appalachia, where access to affordable, healthy food is limited.^4^

Research examining the underlying contextual factors that contribute to adolescents’ dietary behaviors in Appalachia can be used to inform the development of effective obesity-prevention programming.^5^ Few studies have used qualitative methods to examine these factors among adolescents in Appalachia.^5^,^6^ This study seeks to address this gap by examining barriers to healthy eating in this population.

## METHODS

**Figure 1:**
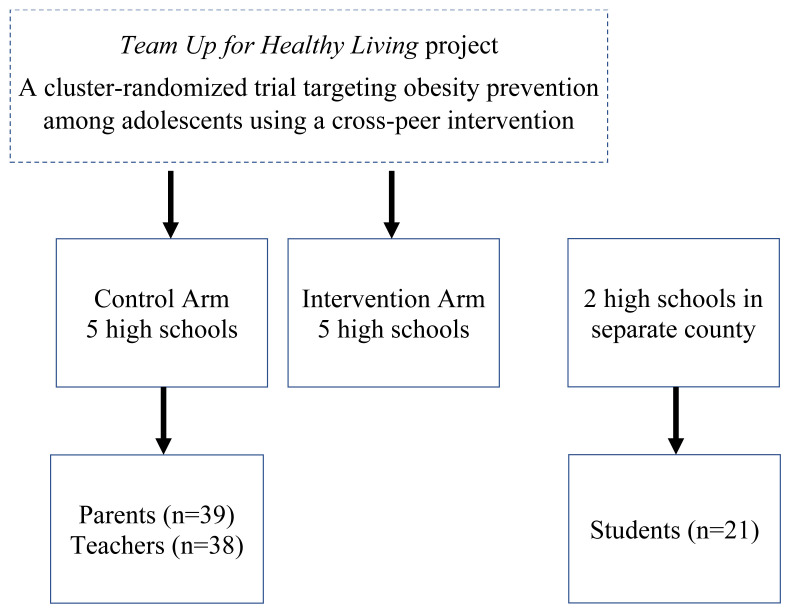
Sampling Frame

During 2013–2014, thirteen focus groups (range, 3–7 participants) and 22 semi-structured interviews were conducted to explore strategies to engage parents in adolescent obesity prevention. The current study focuses on a subset of questions related to barriers of healthy eating (Table 1). Purposive sampling methods were used to recruit participants. Recruitment efforts included flyers and e-invites using school email distribution lists. Participants included parents of adolescents (n=39) and teachers (n=38) from five high schools in the control arm of the *Team Up for Healthy Living* project (NIMHD Grant R01MD006200). High school students (n=21) were recruited from two high schools in a separate contiguous county to avoid biasing the Team Up project (Figure 1; Table 2). Written consents and child assents were obtained prior to beginning the study. College students were trained to conduct interviews. Sessions lasted 30–45 minutes and were audiotaped and transcribed verbatim. Analysis was completed in 2015.

Thematic network analysis (TNA) was used to analyze the text.^7^ TNA uses a multi-stage process which includes: (1) identification of basic themes originating from the coded material, (2) rearranging basic themes into organizing themes, (3) identification of global themes to represent the central points, and (4) analysis of the thematic networks. Themes were compared across participant groups prior to summarizing findings overall. A minimum of two researchers coded the data independently and converged to address any inconsistencies. The study was approved by the Institutional Review Board at East Tennessee State University.

## RESULTS

Analysis of participants’ responses resulted in identification of eight central organizing themes and three global themes: cultural norms, school-based nutrition policy/programming, and rurality (Table 3). The term participant is used to denote the shared opinions of parents, teachers, and students.

### Cultural Norms

#### Beliefs and practices

According to participants, it is difficult for parents to influence the dietary choices of adolescents. Phrases commonly associated with this issue included *no control* and *nothing you can do.* Lack of parental supervision was another concern raised by participants. A teacher stated: *I have kids that come to first period and they’re famished. Nobody’s fed them anything…They can’t learn. They can’t exercise*.” Youth are left to *fend for themselves the majority of the time*, one parent noted.

Most participants identified peer influence as an important factor in shaping adolescents’ dietary behaviors. While some adolescents were making healthy choices and having a positive influence on peers, *pack mentality*, as one parent referred to it, resulted in poor dietary choices. Participants also understood the connection between dietary preferences and community norms: *Cheese, milk, bread, junk, cookies, that’s where my buggy goes…We’ve just been trained, right?* one parent said.

#### Time management

Nearly all participants agreed lack of time limited their ability to eat healthy. Family meals were uncommon, and adolescents were often responsible for preparing food for themselves and younger family members.

#### Preference for unhealthy foods

Participants had a strong preference for unhealthy foods, such as sodas, pizzas, burgers, and sweets. Unhealthy foods were described as having an addictive quality: *You try to put them [youth] on the straight and narrow. They want their drug. Their drug is bad food*, one teacher noted. Teachers described numerous examples of high school students eating unhealthily. One example stood out among others: *I’d have a girl come in and have a big PowerAde and a supersized KitKat. That’s what she ate for breakfast every single morning. She had a day’s worth of calories for breakfast.*

### School-Based Nutrition Policy and Programming

#### Implementation challenges

School enforcement of food policies was recognized as an essential health promotion strategy; however, the school’s actual role in enforcing food policies was debatable. Teachers felt it was not their responsibility to police packed lunches: *If their momma packed it, I don’t care what it is. It’s their momma’s business.* Another commented on students bringing in fast food: *But who’s going to tell a child not to eat. I’m not!* Food policies were not enforced consistently: *They see somebody with a McDonalds^®^ sweet tea they tell them to throw it away. Other days they look straight at you and not say anything*, one student remarked.

Other implementation challenges focused primarily on foods served at lunch. Teachers and parents expressed concern about portion sizes and students commented on food quality. Students described foods as greasy and felt they had limited access to fresh foods. As a result, most students in the study did not eat school lunch. They either ate from vending machines or after school.

#### Limited healthy eating programming

Participants could identify only a few nutrition-focused curricula. Teachers cited lack of confidence *integrat[ing] health into non-health courses.* Other teachers felt it was outside their scope of responsibility or that the school environment defeated class health promotion efforts. These quotes provide context: *As a math teacher, I just don’t see me talking about health. Our primary responsibility is to serve them in the academic areas that we’ve been hired to teach*, and *the unhealthy foods being served in the cafeteria defeated…what you would do in the classroom*.

Other programming issues included lack of snack time during the school day and access to empty-calorie foods via vending machines. Although efforts have been made to enhance the food quality available in vending machines and concessions, there remains a strong reliance on empty-calorie foods: *the drink machines offer nothing but sugarless drinks but they’re right next to the Snickers bars and honeybuns*, one teacher remarked. In most cases, access to vending during lunch was frustrating for teachers and students because it triggered unhealthy choices. *I eat the snack machine. That’s where I go*, one student commented. Participants felt it would be challenging to change the school’s food culture. Parents noted: *We had fruit last year for a while, but it doesn’t sell* and *You’re not going to come to a concession stand and eat a banana over a cheeseburger.*

#### Limited stakeholder engagement

Participants were unaware of any parental involvement in discussions about the school food environment. One parent stated: *We don’t get to give our input.* Other parents felt the schools *were not publicizing what they were doing.* Students also expressed frustration with the top-down approach to decision-making within the school setting.

Most participants, including teachers, were unfamiliar with the process schools used to create local nutrition policies. According to teachers, parents were unsupportive of school nutrition policies. Teachers cited numerous complaints from parents after restrictions were placed on unhealthy foods being brought into schools. Perceptions that parents were unsupportive surfaced only during discussions with teachers.

## Rurality

### Limited socioeconomics

Inadequate resources in the home and community were recognized as having a significant impact on students’ health. Participants believed many parents were unable to provide healthy options to children because healthy foods were more expensive. They also expressed concern for youth who experience chronic food insecurity, emphasizing the central role schools played in alleviating this issue. A teacher responded: *she came to my [class]room and said will they still cook out tomorrow because we don’t have any food at my house right now*. Students’ comments add insight: *this is a really poor county so some kids might not go home and get to eat.*

Participants felt county schools were under-resourced and had limited ability to offer healthy meal options. One parent said: *I went to one meeting and I suggest have a peanut butter sandwich I mean that’d be better…I know the city school offers that and I said why can’t the county*? A student remarked: *we [the school] probably can’t afford all the healthy stuff that we’re requesting.*

### Food deserts/swamps

Participants voiced concerns about the lack of grocery stores and high prevalence of fast-food stores in their communities. Distance to grocery stores created a reliance on convenience stores even though foods were more expensive. Many parents expressed a desire to eat healthier and some were employing better food purchasing habits. However, most parents said their children had access to unhealthy foods at home.

Participants felt that easy access to fast-food stores made them difficult to avoid. Students were observed eating fast food before and after school, noting the long lines at the drive thru. It was not uncommon for students to *eat out every day.* A parent remarked: *It’s impossible for me to resist the urge to just swing through and if it were inconvenient but it’s not.* Many participants believed fast-food outlets targeted low socioeconomic communities, contributing to high rates of obesity, diabetes, and heart disease in the region. Eating healthy also required multiple trips to the grocery store to purchase items with a *shorter shelf life*; therefore, purchasing processed foods was more convenient: *It’s easier to go to McDonalds^®^ and get a Big Mac^®^ than it is to cook*, one parent acknowledged. *It’s a matter of convenience. Here’s your poptart*, a teacher said.

For many participants, cost was the biggest challenge to eating healthy: *It’s more expensive to eat healthy than it is to buy an extra value meal*, one parent commented. Most teachers agreed: *The value meal is much cheaper than the salad.* Taxes on unhealthy foods and discounts on nutritious foods were regarded as possible solutions to reducing food costs: *We’re an obese country…tax the chips more than the fresh produce*, one parent argued. Students made similar observations: *Raise the prices on unhealthy and lower the prices on healthy.*

## STRENGTHS AND LIMITATIONS

The study has several strengths including a large sample size and insights from multiple groups (e.g., parents, teachers, students). Study limitations include the possibility of self-selection bias; however, use of multiple data sources minimized this bias. Qualitative research is subjective in nature. Thus, the analyst can influence the development of themes. To enhance analytic rigor, multiple coders analyzed the data independently and met regularly to confirm findings.

## IMPLICATIONS

This study provides important insights into barriers to healthy eating among adolescents in southern Appalachia and points to future directions for research and practice. Participants discussed how adolescents’ eating behaviors were shaped by social, physical, and environmental factors (e.g., cultural norms, school-based nutrition policy/programming, and rurality). Much of the discussion centered around two environmental factors associated with rurality (e.g., limited socioeconomics and food deserts/swamps). While these factors are often more resistant to change, participants identified several windows of opportunity that school administrators and local officials can consider when developing local health policies. These included expanding stakeholder engagement, supporting local schools, and making healthy eating more affordable. While similar findings have been reported elsewhere,^5^,^6^,^8^ this study is unique because it is one of only a few studies^5^,^6^ to employ qualitative methods to assess barriers to healthy eating among adolescents in Appalachia.

Residents in Appalachia are less likely to meet dietary guidelines and may experience higher levels of food insecurity compared with residents nationally.^9^ Poor eating habits and disparities in obesity rates have also been observed among southern Appalachian youth when compared nationally.^2^,^10^ Appalachian youth may experience greater difficulty changing eating habits than non-Appalachians,^11^ due to cultural, economic and geographic barriers.^12^ The findings from the current study can be used by school health personnel to develop ecologic approaches^5^,^12^ to school-based health promotion programming in the region. Student associations can also use these findings to actively participate in efforts to improve school food offerings.

**SUMMARY BOX**

**What is already known about this topic?** Dietary habits can be difficult to modify in adolescents, particularly in Appalachia, where access to affordable healthy foods is limited.

**What is added by this report?** This study describes the complex and challenging role that individual, home, school, and community environments have on healthy eating among adolescents and how these findings can be used by school health personnel to develop ecologic approaches to school-based health promotion programming in the region.

**What are the implications for public health practice, policy, and research?** These findings provide important insights into barriers to healthy eating in this population and point to future directions for research and practice. For example, the findings can be used by school personnel to develop ecologic approaches to school-based nutrition programming in Appalachia. Student associations can also use these findings to actively participate in efforts to improve school food offerings.

## Figures and Tables

**Table 1:** Sample Interview Questions

Sample Interview Questions
What does it mean to you to eat healthy?
How can we encourage healthy eating among high schoolers?
How does the high school encourage healthy eating among high schoolers?
What role should the school play in encouraging healthy habits among high schoolers?
What role should parents and families play in encouraging healthy habits among high schoolers?
How can we encourage high schools to be more involved in promoting healthy habits among high schoolers?
How can we encourage parents and families to be more involved in promoting healthy habits among high schoolers?
Discuss some ways we could involve parents in programs to encourage healthy eating and physical activity in their children.

**Table 2:** Sample Characteristics

	Parents (n = 39) n (%)	Teachers (n = 38) n (%)
Age, y, mean (SD)	45.23 (8.1)	44.95 (11.2)
**Gender**		
Female	31 (79.5)	29 (76.3)
Male	8 (20.5)	9 (23.7)
**Highest Level of Education**		
Less than high school	1 (2.6)	-
High School or GED	11 (28.95)	2 (5.3)
Some college	11 (28.95)	2 (5.3)
College degree	15 (39.5)	34 (89.4)
**Years in Region**		
0 to 10 Years	6 (15.4)	7 (18.4)
11 to 20 years	5 (12.8)	4 (10.5)
More than 20 years	28 (71.8)	27 (71.1)
**Employment Status**		
Full Time	21 (53.8)	38 (100.0)
Part Time	5 (12.8)	-
Self Employed	1 (2.6)	-
Unemployed	7 (18.0)	-
Other	5 (12.8)	-
**Children in Elementary or Middle School**		
I do not have children	-	7 (18.4)
None	20 (51.3)	21 (55.3)
1 or more children	19 (48.7)	10 (26.3)
**Children in High School**		
I do not have children	-	7 (18.4)
None	-	26 (68.4)
1 or more children	39 (100.0)	5 (13.2)

NOTE: Gender (13 females, 8 males) was the only demographic information collected on students.

**Table 3:** Barriers to Healthy Eating

Global Themes	Organizing Themes
Cultural norms	Beliefs and practices
	Time management
	Preference for unhealthy foods
School-based nutrition policy and programming	Implementation challenges
	Limited healthy eating programming
	Limited stakeholder engagement
Rurality	Limited socioeconomics
	Food deserts/swamps
